# Der Zentrumbereichskern Wilmersdorfer Straße in Berlin-Charlottenburg in Zeiten multipler Krisen – gestärkt durch Nutzungsmischung, Kiezcharakter und Kuration?

**DOI:** 10.1007/s00548-023-00866-x

**Published:** 2023-06-07

**Authors:** Lech Suwala, Nina Pfeil, Max Lange, Linus Pfeiffer, Hans-Hermann Albers

**Affiliations:** Institut für Stadt- und Regionalplanung, Fachgebiet Stadt- und Regionalökonomie, Technische Universität Berlin, Hardenbergstr. 40a, 10623 Berlin, Deutschland

**Keywords:** Einzelhandel, Stadtplanung, Gewerbliche Erdgeschosse, Geschäftsstraßenmanagement, Berlin City-West, Retail, Urban planning, Ground-floor commerce, High street management, Berlin City West

## Abstract

Die Coronapandemie hat zwischenzeitlich für einen durchschnittlichen Rückgang der Passantenfrequenzen von bis zu 40 % in den Hauptgeschäftsstraßen der deutschen Großstädte gesorgt. In Kombination mit den Schreckgespenstern Onlinehandel, der zunehmenden Einzelhandels- und Filialdichte und neuerdings der Inflation kommen besondere Herausforderungen auf innerstädtische Geschäftsstraßen und ihre Erdgeschosseinheiten zu. Dieser Beitrag setzt sich mit dem Zentrumbereichskern Wilmersdorfer Straße in Berlin-Charlottenburg auseinander und hat das Ziel, zu zeigen, dass Nutzungsmischung, Kiezcharakter und Kuration im Verbund innerstädtischen Geschäftsstraßen und ihren Erdgeschosseinheiten gerade in Krisenzeiten helfen können. Dies geschieht basierend auf einem sozio- und regionalökonomischen Profil der Geschäftsstraße, das sich aus lokalen sekundärstatistischen Daten, einer Kartierung der Liegenschaften und 13 Expertengesprächen mit mannigfaltigen Stakeholdern speist. Es kann gezeigt werden, dass ein breit gefächerter Nutzungs- und Funktionsmix jenseits von Handelsaktivitäten mit einer integrierten Nahversorgungsfunktion, gepaart mit Kiezcharakter und einer Kuration, die offen für neuartige institutionelle Verbünde mit der Zivilgesellschaft ist, innerstädtische Geschäftsstraßen sehr widerstandsfähig machen kann. Dies gilt insbesondere dann, wenn die Geschäftsstraße zusätzlich in einen Kiez eingebettet ist, auf einen räumlich verwurzelten Kundenstamm trifft und eben auf dessen lokaleren Lebensradien (z. B. als gesellschaftlicher Treffpunkt) Bezug nimmt.

## Abstract (en)

The COVID-19 pandemic has caused an average decline in pedestrian footfall of up to 40% within the main commercial streets of major German metropoles. Combined with online retail, increasing retail and branch densities, and, more recently, inflation, inner-city commercial streets and their ground-floor units face pertinent challenges. This article examines the high street Wilmersdorfer Straße in Berlin-Charlottenburg and aims to illustrate that a mix of (non-)commercial uses, community appeal, and curation combined can support inner-city commercial streets and their ground-floor units, especially in times of crisis. The analysis is based on a socio-economic and regional profile of the commercial street, which is informed by statistical data, a mapping of properties, and 13 expert interviews with a variety of stakeholders. It can be demonstrated that a diversified mix of uses and functions beyond retail activities with an integrated local supply, coupled with community appeal and curation that is open to novel institutional alliances with civil society, can make inner-city commercial streets resilient. This is especially true if the commercial street is also embedded in a neighborhood, meets a spatially rooted client base, and refers to their more local life realities (e.g., as a social gathering spot) as a consequence of the crisis.

## Einführung

Die Wilmersdorfer Straße ist eine historisch gewachsene und in Teilen als Fußgängerzone verlaufende Geschäfts- und Einkaufsstraße im Westen Berlins, die oft im Schatten der Tauentzienstraße (rund um die Kaiser-Wilhelm-Gedächtnis-Kirche unweit des Bahnhofs Zoologischer Garten) und des Kurfürstendamms steht. Daran änderte lange Zeit weder ihre Zugehörigkeit zu zwei Zentrumsbereichskernen (höchste Zentralitätsstufe des städtischen Stadtentwicklungsplanes) der City-West noch ihre 1a-Lage in Berlin nichts (SenSW 2019). Zudem wurde ihr ein in die Jahre gekommenes und verstaubtes Image (z. B. Mangel an Aufenthaltsqualität, zusammengewürfelte Architektur, unattraktive Außenraumgestaltung, einfache Gastronomieangebote) nachgesagt. Zu groß schien die Konkurrenz durch langfristige Trends wie noch zentralere Lagen, moderne Malls, die Ausschöpfung von Flächenpotenzialen im Einzelhandel und den Onlinehandel. Gleichzeitig mach(t)en kurzfristige Zwangslagen, wie Kontaktbeschränkungen der Coronapandemie sowie der daraus resultierende Rückgang der Passant*innenfrequenz, das Inflationsgeschehen und die schlechte Verbraucher*innenstimmung seit dem Ukraine-Krieg, Geschäftsstraßen und insbesondere deren gewerblichen Erdgeschosseinheiten zu schaffen (Handelsverband Deutschland 2022; Statistisches Bundesamt 2022). Wie steht es nun um die Wilmersdorfer Straße? Werden innerstädtische Geschäftsstraßen als Konsumzentren ein Opfer von Corona und Homeoffice wie es in aktuellen Studien heißt (Alipour et al. 2022; Appel und Hardaker 2022)?! Wie kann weitergedacht, diversifiziert und interveniert werden? Können Innenstädte samt Geschäftsstraßen und gewerblichen Erdgeschosseinheiten als neue Orte der Begegnung gestärkt aus dem Zeitalter des Homeoffice und neuer lokaler Lebensradien hervorgehen?

Dieser Beitrag greift ebendiese Fragen auf und betrachtet die Wilmersdorfer Straße in Berlin-Charlottenburg als Geschäftsstraße im Allgemeinen und deren gewerbliche Erdgeschosseinheiten im Besonderen. Er hat das Ziel zu zeigen, dass Nutzungsmischung, Kiezcharakter und Kuration im Verbund innerstädtischen Geschäftsstraßen und ihren Erdgeschosseinheiten gerade in Krisenzeiten helfen können. Dies erfolgt auf der Basis eines sozio- und regionalökonomischen Profils der Wilmersdorfer Straße, das sich aus lokalen sekundärstatistischen Daten sowie einer lückenlosen Bestandsaufnahme und Kartierung der gewerblichen Erdgeschosseinheiten speist. Ferner wurde das Profil im Rahmen mehrerer strukturierter Ortsbegehungen und eines Stimmungsbildes, bestehend aus 13 Expert*innengesprächen mit unterschiedlichen Stakeholdern zu Nutzungen, Steuerungsoptionen und zukünftigen Maßnahmen (Dauer jeweils 45 bis 60 min), angereichert. Die Auswahl der Stakeholder wurde mit dem Ziel durchgeführt, ein möglichst breites Spektrum an Perspektiven abzudecken und setzte sich aus Vertreter*innen des Senats und des Bezirks (Politik und Verwaltung), aus (un‑)abhängigen Gewerbetreibenden (Inhaber*innen und Filialist*innen, Wirtschaft) sowie aus lokalen Initiativen (Zivilgesellschaft) zusammen. Grundsätzlich war die fünfmonatige Untersuchung (Mai bis September 2022) auf die gewerblichen Erdgeschosseinheiten entlang der Wilmersdorfer Straße auf ihrer gesamten Länge von 1,9 km beschränkt. Gleichzeitig wurde eine Betrachtung der Umgebung, d. h. der Obergeschosse der untersuchten Liegenschaften, der Seiten- und querenden Hauptverkehrsstraßen sowie der umgebenden lebensweltlich orientierten Räume, integriert. Hierfür wurden ergänzend einschlägige Abschlussarbeiten und Gutachten konsultiert, um zielgerichtete, passgenaue und kontextspezifische Aussagen für die Geschäftsstraße als solches treffen zu können (z. B. Acocella et al. 2021; Pfeil 2021; Suwala et al. 2022).

## Geschäftsstraßen, gewerbliche Erdgeschosseinheiten und stationärer Einzelhandel in der (post‑)pandemischen Stadt

Seit geraumer Zeit lassen sich in der einschlägigen Literatur Diskurse beobachten, die versuchen auf die oben genannten langfristigen Trends Antworten zu finden. Dazu werden überinstitutionelle Verbünde (z. B. mit der Zivilgesellschaft) erforscht, mehr privatwirtschaftliche Verantwortung beim Ausbau von Geschäftsstraßen gefordert oder neue Formen der Funktionsmischung bei der Revitalisierung von Geschäftsstraßen vorgeschlagen. Ferner wird der Einsatz moderner Technologien und virtueller Welten als smarte oder alternative Lösungen zur Ausgestaltung von Innenstädten samt Geschäftsstraßen, gewerblichen Erdgeschosseinheiten und dem stationären Einzelhandel ins Gespräch gebracht (Henckel et al. 2008; Albers und Suwala 2018; Suwala et al. 2021; Carmona 2022).

Beflügelt wurden diese Diskussionen neuerdings durch eine theoretische und praktische Debatte rund um die (post‑)pandemische Stadt im Allgemeinen (z. B. Bentlin et al. 2021; Klemme 2022) und um kuratierte Erdgeschosse im Besonderem, die als Zugpferde einer Quartiersentwicklung, Stabilisatoren von Geschäftsstraßen oder Aktivitätsanker in (Neubau‑)Quartieren diskutiert werden (z. B. Pfeil 2021; Rochholl 2021; Appel und Hardaker 2022). In diesem Zusammenhang fördert das Bundesministerium des Inneren im Rahmen der Nationalen Stadtentwicklungspolitik 13 Pilotprojekte von Kommunen, Vereinen, Initiativen, Unternehmen und anderen Akteur*innen, die mit lokalen Wirtschaftskreisläufen, neuartigen institutionellen Verbünden oder integrierten Stadtentwicklungsstrategien unter Resilienzaspekten, Fragen auf neue Lebenswirklichkeiten im öffentlichen Raum angehen sollen. Diese Gemeinschaftsinitiative von Bund, Ländern und Kommunen ergänzt damit die laufenden Bundesprogramme der Städtebauförderung, wie z. B. das Programm lebendige Zentren (BBSR 2020, 2021). Dabei fungiert die Berliner Senatsverwaltung für Stadtentwicklung, Bauen und Wohnen als Projektträgerin eines Vorhabens dieser Gemeinschaftsinitiative, bei dem innovative Ansätze zu kuratiertem Management von Handelsflächen in Erdgeschosszonen etablierter Zentren ausprobiert werden sollen. Die konkrete Umsetzung soll dieses und nächstes Jahr in der Hauptstadt im Rahmen des Mittendrin Berlin!-Programms pilothaft erprobt werden (Kruse und Plate 2022). An dieser Schnittstelle setzt der Beitrag an und versucht zu zeigen, dass Geschäftsstraßen mit einem Dreiklang aus Nutzungsmischung, Kiezcharakter und Kuration insbesondere in Erdgeschosseinheiten krisenfest gemacht werden können.

## Die Wilmersdorfer Straße – Geschichte, Profil und räumliche Differenzierung

**Abb. 1:**
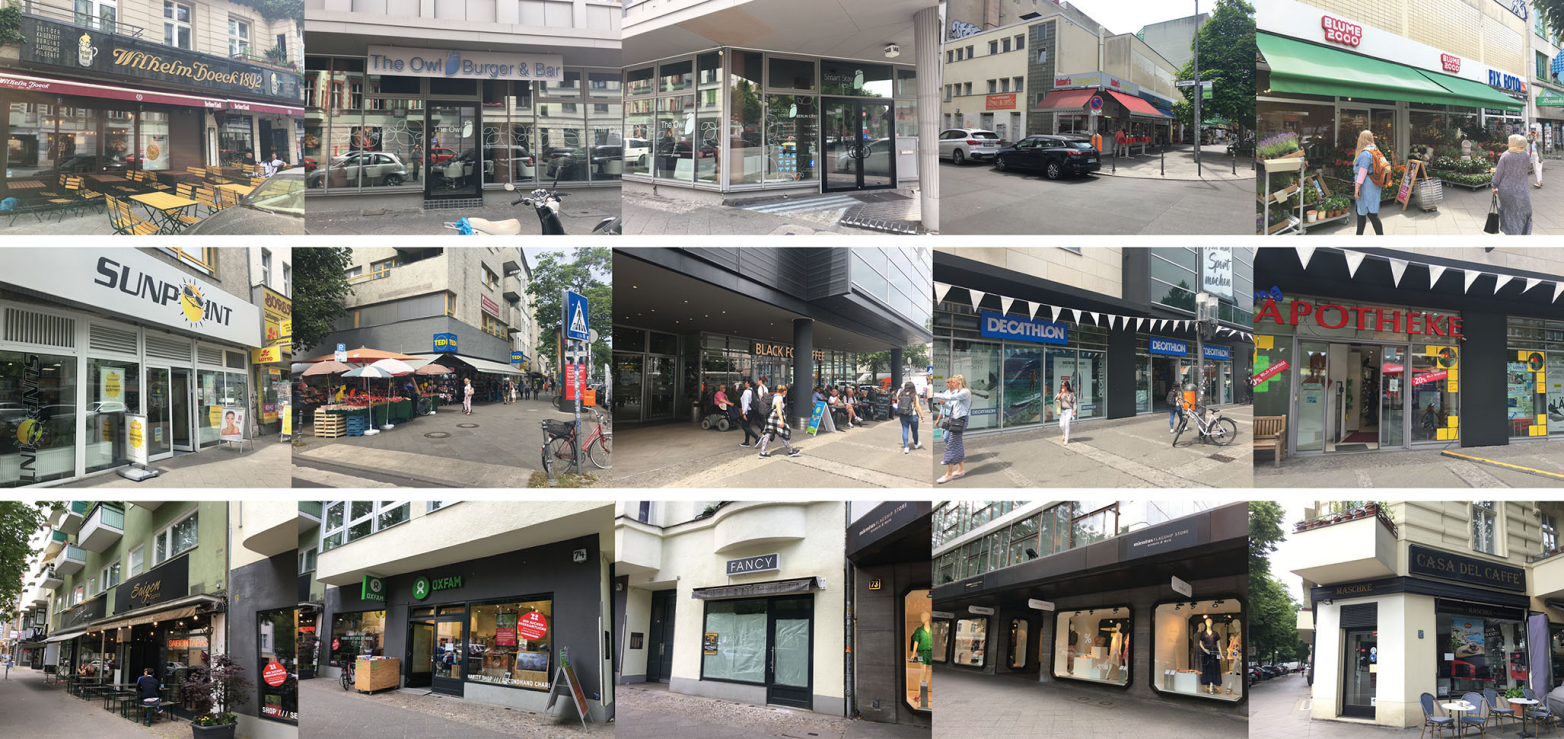
Bildstrecken zur gewerblichen Erdgeschossnutzung in der Wilmersdorfer Straße (*oben*, nördlicher Teil; *mittig*, mittlerer Teil; *unten*, südlicher Teil). (Eigene Bilder)

**Abb. 2:**
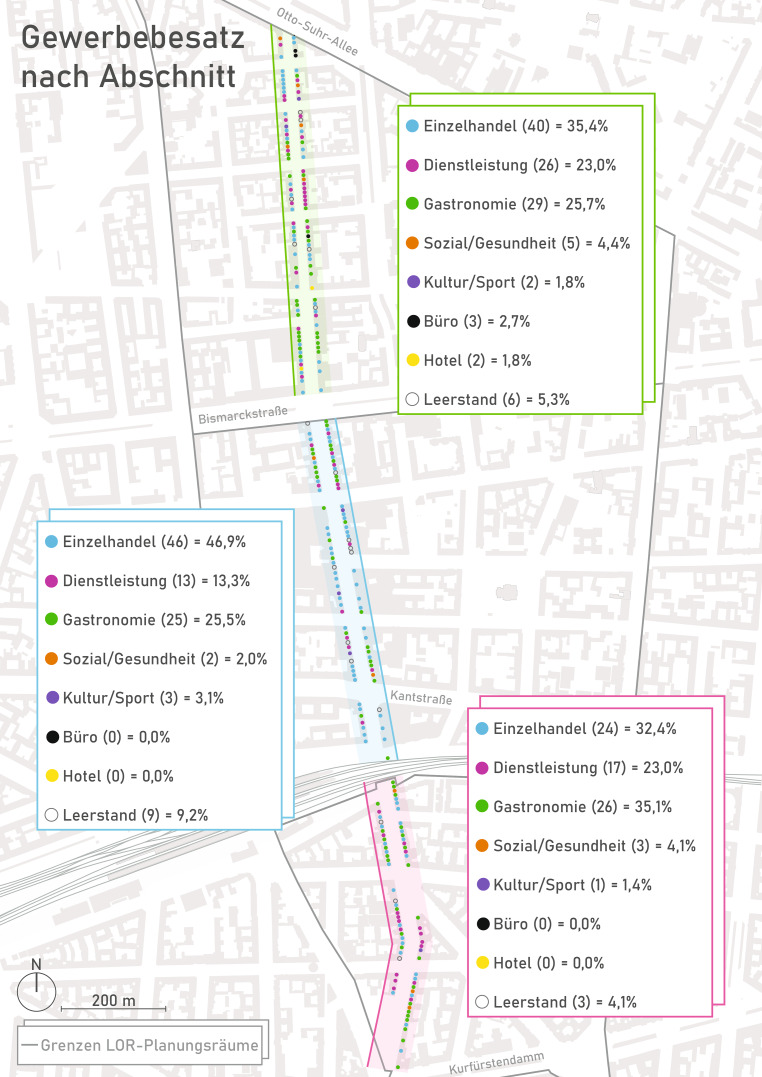
Branchenstruktur – differenziert nach Teilbereichen (Nord, Mitte, Süd) entlang der Wilmersdorfer Straße. (Eigene Darstellung)

Die Geschichte der Wilmersdorfer Straße als bürgerliche Geschäftsstraße reicht bis in die zweite Hälfte des 19. Jahrhunderts zurück. Neben der planmäßigen Anlage einer gründerzeitlichen Blockrandbebauung (Pläne von James Hobrecht, ab 1862) und des Bahnhofbaus von Charlottenburg (1882) wurde die Entwicklung durch die Gründung des Warenhauses Graff & Heyn, als Vorgänger des heutigen Kaufhauses der Galeria Karstadt Kaufhof GmbH, maßgeblich beflügelt (Pfeil 2021, S. 56). Die Wilmersdorfer Straße, und hier ist gleichzeitig das Untersuchungsgebiet gemeint, erstreckt sich beidseitig der namensgebenden Straße, in Teilen davon als Fußgängerzone, und umfasst die gewerbliche Erdgeschossnutzung (vgl. Abb. 1) auf einer Länge von knapp zwei Kilometern zwischen dem Abzweig Wilmersdorfer Straße/Otto-Suhr-Allee im Norden und der Mündung der Wilmersdorfer Straße in die Lewishamstraße am Adenauerplatz bzw. Kurfürstendamm im Süden (vgl. Abb. 2).

Zerstörungen im Zweiten Weltkrieg, insbesondere zwischen der Bismarckstraße und der Stadtbahn (heutige Fußgängerzone, mittlerer Teil, vgl. auch Abb. 1), hatten erhebliche Auswirkungen auf das bauliche Ensemble der Geschäftsstraße. Diese Auswirkungen resultierten in einem architektonischen Flickenteppich aus unterschiedlichen Geschosshöhen sowie Fassadengestaltungen und bescherten der Straße ihre bis in die Gegenwart bestehende Dreiteilung in einen südlichen (Kurfürstendamm bis zur Stadtbahn, in rot), mittleren (Stadtbahn bis Bismarckstraße, in blau) und nördlichen Abschnitt (Bismarckstraße bis Otto-Suhr-Allee, in grün) (Weißpflug et al. 2005, vgl. Abb. 1 und 2). Diese Nachkriegsmoderne prägte ab den 1970er-Jahren aber auch ein zunehmend monofunktionales Bild – trotz einer U‑Bahn-Anbindung und Umwandlung der Straße in eine Fußgängerzone (1978) – und brachte die Geschäftsstraße insbesondere in den 1990er-Jahren zunehmend ins Hintertreffen, als der Fokus auf die Etablierung einer leistungsfähigen Einzelhandelsstruktur in Ostberlin und nichtintegrierte Standorte in verkehrsgünstigen Lagen des Berliner Speckgürtels gelegt wurde. Erst mit der Gründung der einzelhandelsspezifischen Arbeitsgemeinschaft Wilmersdorfer Straße (2001) sowie den Eröffnungen des Kant-Centers (2005) und der Wilmersdorfer Arcaden (2007) gab es „im Westen wieder was Neues“ (Lange et al. 2011; Pfeil 2021).

Planerisch, baulich, verkehrstechnisch und sozioökonomisch lässt sich diese Dreiteilung sehr gut nachvollziehen. Planungsrechtlich gehören der mittlere und der südliche Teil der Wilmersdorfer Straße zu zwei Zentrumsbereichskernen der City-West, während der nördliche Teil keiner Hierarchiestufe zugeordnet wird und im Norden an das Nahversorgungszentrum Richard-Wagner-Platz/Otto-Suhr-Allee rund um das Rathaus Charlottenburg grenzt. Verkehrstechnisch ist die Wilmersdorfer Straße in ein enges Geflecht aus querverlaufenden Haupt- und Nebenverkehrsadern (z. B. Bismarckstraße, Kantstraße, Stadtbahn, Kurfürstendamm) eingebettet, die im Endeffekt diese Dreiteilung durch ihre fragmentierende Wirkung unterstützen. Ferner zeigt die Geschäftsstraße ein sozioökonomisches Nord-Süd-Gefälle entlang von drei, mit der vorgeschlagenen Dreiteilung identischen, lebensweltlich orientierten Räumen, die mit zunehmender Nähe zum Kurfürstendamm zwar wohlhabender (Kaufkraft), aber auch betagter (Altersstruktur), alteingesessener (Wohndauer) und weniger bunt (Migrationshintergrund) bezogen auf einschlägige Vergleichswerte werden. Der unmittelbare Einzugsbereich der Geschäftsstraße umfasst über 50.000 Einwohnende und mittelbar (City West) – je nach Abgrenzung – etwa 300.000–500.000 Einwohnende (SenSBW 2021).

## Nutzungsmischung – Versorgungsfunktion/Sortiment und Branchenstruktur

Die Nutzungsmischung wird im Folgenden durch die Versorgungsfunktion bzw. das Sortiment sowie die Branchenstruktur analysiert. Ein Zentrumsbereichskern (ZBK) suggeriert per Definition neben einer überörtlichen Versorgungsfunktion, einen vollständigen zentrenrelevanten Branchen- und Vertriebsformenmix des Einzelhandels sowie ein Angebot an Gastronomie, Dienstleistungen, Kultur und öffentlichen Einrichtungen (SenSW 2019). Diese Versorgungsfunktion erfüllt die Wilmersdorfer Straße in ihrer Gänze als funktional starkes Zentrum sowohl durch ein umfassendes Angebot in allen Bedarfsbereichen (Sortimentsbreite) als auch durch ein überwiegend mittleres Angebotsniveau (Sortimentstiefe). Wird nur der mittlere Teil der Straße betrachtet, sind zwei Drittel dieses Sortiments auch zentrenrelevant. Während im südlichen Teil ebenso das zentrenrelevante Sortiment knapp überwiegt, hat im nördlichen Teil die Nahversorgungsfunktion die Oberhand (vgl. Acocella et al. 2021, S. 124). Werden sämtliche 285 Gewerbeflächen in den Erdgeschossen über die ganze Länge der Geschäftsstraße bezogen auf die Branchenstruktur betrachtet, fällt zunächst einmal ein Überhang an Geschäften mit Einzelhandel und Gastronomie auf, die etwa zwei Drittel aller Erdgeschosseinheiten ausmachen. Darüber hinaus ist fast jedes fünfte Geschäft der sehr diversen Kategorie Dienstleistungen (58 Geschäfte, wie z. B. Friseur, Reisebüro, Änderungsschneiderei, Nagelstudio, Schlüsseldienst, usw.) zuzuordnen. Mit einer Leerstandsquote von knapp über sechs Prozent (18 Einheiten) steht die Wilmersdorfer Straße nach zwei Jahren Pandemie sehr gut dar. Solche Werte bewegen sich im Rahmen des herkömmlichen fluktuativen Leerstandes (vgl. Interviewpartner*innen [IP] 11, 13). Wird die Branchenstruktur separat in den drei Teilbereichen Nord, Mitte und Süd betrachtet, ergeben sich nicht weiter verwunderlich, strukturelle Merkmale, die mit der Ausstattung des zentrenrelevanten Sortiments – wie dem hohen Anteil des Einzelhandels im mittleren Teil – zulasten anderen Kategorien einhergehen (vgl. Abb. 2). Einrichtungen aus den Bereichen Soziales/Gesundheit (10 Liegenschaften) und Kultur/Sport (6 Liegenschaften) spielen zumindest in Erdgeschossflächen keine nennenswerte Rolle. Diese Kategorien nehmen samt des Hotel‑/Tourismusgewerbes und höherwertigen Dienstleistungen (Arztpraxen, (Werbe‑)Agenturen, Rechtsanwälte) in den Seitenstraßen und/oder Obergeschossen sowie Hinterhäusern einen größeren Stellenwert ein. Damit unterscheidet sich die Wilmersdorfer Straße erheblich von innerstädtischen Geschäftsstraßen, die weitaus höhere Anteile von zentrenrelevanten Sortimenten besitzen, wie z. B. dem Kurfürstendamm mit 73 % (mit Nebenstraßen) und der Tauentzienstraße mit sogar über 90 % (Acocella et al. 2021, S. 120). Obwohl wir mangels Daten keine Aussagen über Entwicklungstendenzen oder coronabedingte Veränderungen hinsichtlich dieser Parameter in den letzten Jahren treffen könnten, kann dennoch folgendes festgestellt werden. Während bspw. die geringeren Anteile der zentrenrelevanten Sortimente in der Vorpandemiezeit grundsätzlich als eine Strukturschwäche von hochrangigen Geschäftsstraßen ausgelegt wurde, zeichnet sich gerade die lokale Versorgungsfunktion in der gegenwärtigen Homeoffice-Realität als ein stabilisierender Anker aus.

## Kiezcharakter – Magnetbetriebe, Betriebsformen, Identität

**Abb. 3:**
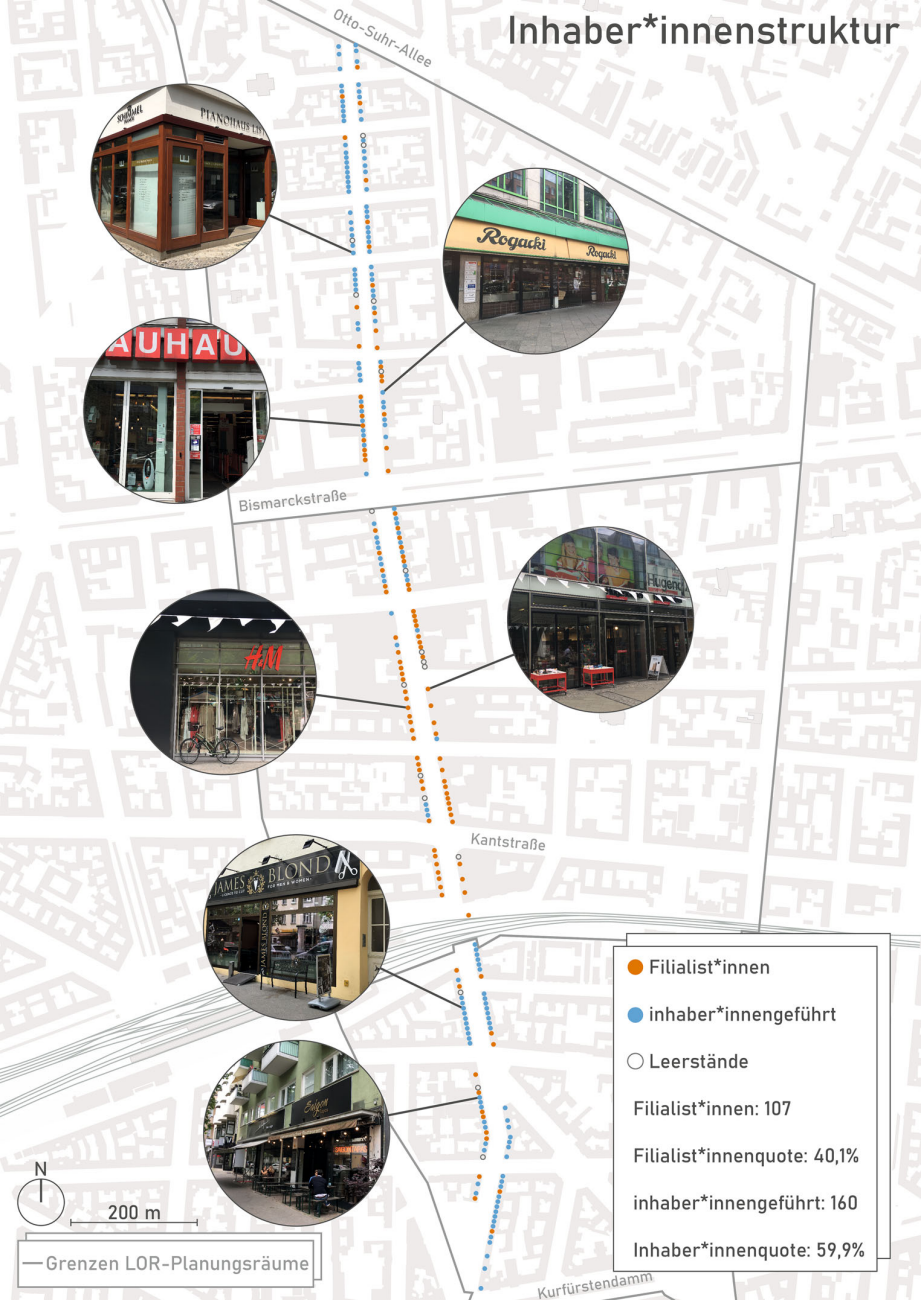
Betriebsformen – differenziert nach inhaber*innengeführten Geschäften und Filialist*innen entlang der Wilmersdorfer Straße. (Eigene Darstellung)

Der Kiezcharakter soll näherungsweise durch Betriebsformen, Magnetbetriebe und O‑Töne der Stakeholder bestimmt werden. Eine oftmals vernommene Aussage „Die Wilmersdorfer Straße ist beides, sowohl eine Kiezstraße als auch eine Geschäftsstraße. Das unterscheidet sie von anderen, reinen Geschäftsstraßen“ (IP11, in Ansätzen aber nicht wortwörtlich IP 1–4, 8–9, 13) lässt sich empirisch auch mit Zahlen unterlegen. Von den 267 aktiven, eigens kartierten Gewerbeeinheiten im Erdgeschoss waren in etwa 60 % inhaber*innengeführte Geschäfte und 40 % Filialist*innen (vgl. Abb. 3). Erfolgt eine Differenzierung nach den drei Teilbereichen, so ergibt sich eine Dominanz der Filialist*innen – etwa drei Viertel aller Erdgeschossnutzungen – im mittleren Teil der Geschäftsstraße, die größtenteils mit der Fußgängerzone identisch ist, während im nördlichen und südlichen Teil inhaber*innengeführte Läden mit genau umgekehrten Vorzeichen (75 % bzw. 79 %) deutlich den Geschäftsbesatz prägen. Im Vergleich dazu ist der Filialisierungsgrad in anderen 1a-Lagen Berlins oder anderer deutscher Großstädte (z. B. München und Hamburg mit zwischen 80 und 90 %) (DZ Hyp 2020, S. 16) mit dem mittleren Teil der Wilmersdorfer Straße vergleichbar. Dies gilt insbesondere dann, wenn sämtliche Gewerbeflächen in den beiden Shopping-Malls (Wilma, Kant Center) mit einbezogen worden wären. Am Ende macht es aber die Mischung und diese sollte lokal gut sortiert sein. Es wurde an anderer Stelle festgestellt, dass in den querverlaufenden Seiten- (z. B. Pestalozzistraße) und Durchgangsstraßen (z. B. Kantstraße) ebenso inhaber*innengeführte Geschäfte dominieren und die eigentliche Geschäftsstraße ergänzen und bereichern (vgl. Acocella et al. 2021, S. 123). Zu dieser Identitätsbildung tragen maßgeblich auch Magnetbetriebe bei, die in der Wilmersdorfer Straße eine doppelte Funktion ausfüllen – zum einen als Frequenzbringer, zum anderen als gesellschaftliche Treffpunkte. Diese Betriebe sind unterschiedlich ausgestaltet, was Größen, Betriebsformen, Zielgruppen und Sortimente angeht: vom klassischen Warenhaus über den familiengeführten Feinkostladen bis hin zur modernen Shopping Mall. Eines aber verbindet diese Betriebe – sie sind (zumeist) „alteingesessen“, räumlich verankert, stark frequentiert und sie generieren somit Alleinstellungsmerkmale. Damit gelten sie sowohl als das Rückgrat als auch Hauptmotive zum Besuch der Geschäftsstraße, indem sie mit vielfältigen Wegeketten und Kopplungskäufen in anderen Läden kombiniert werden können (vgl. auch Kulke 2014). So ist es nicht weiter verwunderlich, dass der Betreiber einer identitätsstiftenden Shopping-Mall folgendes attestiert: „der Großteil unserer Kunden, also 40–45 % kommen aus dem Kiez, also aus der direkten Umgebung“ (IP3).

## Kuration – Steuerungsoptionen und institutionelle Verbünde

**Abb. 4:**
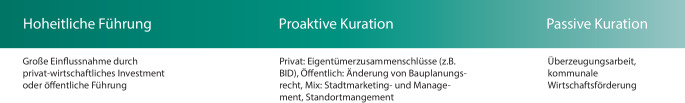
Bandbreite von Kurations- und Steuerungsoptionen für die Wilmersdorfer Straße. (Eigene Darstellung)

Die Nutzungsmischung und der Kiezcharakter sind nur zwei Bausteine, um Geschäftsstraßen in Zeiten multipler Krisen widerstandsfähig zu machen. Auf die Zusammenführung und Steuerung vorhandener Strukturen kommt es an. Neudeutsch wird das als Kuration (vgl. für eine detaillierte etymologische und inhaltliche Auseinandersetzung bezogen auf Erdgeschosszonen, sh. Rochholl 2021) bezeichnet. Am Beispiel von Steuerungsoptionen sollen einige Vorschläge zur Kuration, auch außerhalb hoheitlicher Führung, aufbauend auf den beschriebenen vorhandenen Strukturen, den erhobenen Daten und den Interviews formuliert werden. Grundsätzlich bringen sich bereits mannigfaltige Akteur*innen in unterschiedliche Aktivitäten der Wilmersdorfer Straße ein und übernehmen dabei in Teilen Steuerungsaufgaben. Zu den wichtigsten gehören die AG Wilmersdorfer Straße, ein Zusammenschluss aus Gewerbetreibenden, der seit nunmehr 20 Jahren unterschiedliche Initiativen, wie bauliche und infrastrukturelle Aufwertungsmaßnahmen und die temporäre Bespielung der Fußgängerzone, im Stile einer typischen Interessensgemeinschaft übernimmt sowie die bezirkliche Wirtschaftsförderung, die hier im Hintergrund durch zahlreiche Informationskampagnen, Veranstaltungen und Förderprogramme (z. B. Etablierung eines Standortmanagements) einwirkt (IP 4, 9, 11, 13). Diese „traditionelle Akteurslandschaft“ ist durch zivilgesellschaftliche Initiativen wie die Nachbarschaftsinitiative Karl-August-Kiez zu ergänzen, die bereits jetzt in und rund um die Geschäftsstraße aktiv sind. Dabei lässt sich ein breites Kontinuum an Möglichkeiten der Kuration gemäß der Stakeholderaussagen aufspannen (vgl. Abb. 4). Ausgehend von einer passiven Kuration mit vornehmlich persuasiven Elementen der Vernetzung und Kommunikation könnte ein zivilgesellschaftliches Engagement mit Selbstverpflichtungen, lokal autarke Einheiten (sh. Handlungsempfehlungen) befördern. Eine proaktive Kuration könnte seitens des gegenwärtigen Standortmanagements, einer Interessens- oder Immobiliengemeinschaft, wie z. B. der AG Wilmersdorfer Straße – allerdings mit Einbezug der Zivilgesellschaft gedacht –, ausgehen und konkrete Schritte (z. B. Erweiterung der Fußgängerzone) auch mit rechtlichen und finanziellen Instrumenten untermalen. Über Vorbilder aus Hamburg oder der City West, die bereits über Erfahrungen mit Business Improvement Districts haben, wäre nachzudenken (Schote 2020). Schließlich wären Public-Private-People-Partnerships (PPPP) unter hoheitlicher Führung (z. B. des Bezirks) vorstellbar, die systemische Anliegen und Strategien für die ganze Straße, ggf. durch einen Fonds für kommunalen Flächenerwerb, unter Beteiligung sämtlicher Stakeholder und hier insbesondere auch der Eigentümer*innen vorantreiben.

## Handlungsempfehlungen und Fazit

Aufbauend auf den Erläuterungen zu Nutzungsmischung, Kiezcharakter und Kuration favorisieren unsere Handlungsempfehlungen, auch auf der Basis der Stakeholderaussagen, insbesondere im Norden und Süden der Geschäftsstraße lokale und autarke Funktionseinheiten aus einem Anker- oder Magnetbetrieb, um die sich in Laufnähe (5 min Fußweg) viele weitere Nutzungen, wie Gastronomie, Unterhaltung und Kultur, soziale oder öffentliche Einrichtungen, Werkstätten (Co–Working, Labs) oder kleine Betriebe urbaner Produktion, befinden und vordergründig die angrenzenden Kieze versorgen. Im Grunde sind das alles Maßnahmen zum Identitätsaufbau im Sinne eines Community Buildings. Bezogen auf den Kernbereich der Fußgängerzone gilt es, intelligent zu diversifizieren und dabei den monostrukturierten und filialistenbasierten Einzelhandel durch den Einzug bezirklicher Einrichtungen (z. B. Bibliothek, Familienzentrum), eine (temporäre) Eventisierung (z. B. Pop-ups, Straßenfeste) und ein Showrooming (z. B. Games Events, Gläserne Manufaktur lokaler Unternehmen als Rekrutierungsstrategie) zu unterstützen. Die ergänzenden Funktionen der Geschossflächen darüber und der Seiten- und Hauptverkehrsstraßen dürfen dabei nicht übersehen werden. Zahlreiche Beispiele dazu wurden in mannigfaltigen deutschen Städten bereits erprobt (BBSR 2021). Besondere Sorgfalt ergibt sich bei identitätsstiftenden Magnetbetrieben – sollten diese in eine Schieflage geraten, sind hier alle Stakeholder gefordert, um nachhaltige Lösungen zu eruieren. Hoheitliche Träger müssen in diesen Fällen mit privatwirtschaftlichen Unternehmen und ggf. Eigentümer*innen Möglichkeiten ausloten, um strategischen Leerstand zu vermeiden. Seit der abermaligen Galeria Karstadt Kaufhof-Krise sind hier innovative Lösungen, wie z. B. neue Konzepte, die Einzelhandel mit anderen Funktionen (Verwaltung, Freizeit, Bildung) auf diesen Flächen verbinden, gefragt.

Es konnte gezeigt werden, dass innerstädtische Geschäftsstraßen mit einem breit gefächerten Nutzungs- und Funktionsmix jenseits ihrer Handelsaktivitäten und einer integrierten Nahversorgungsfunktion, gepaart mit Kiezcharakter und einer Kuration, die offen für neuartige institutionelle Verbünde (PPPP) sind, besonders widerstandsfähig sein können. Dies gilt insbesondere auch dann, wenn ein organisch gewachsener Baubestand auf einen räumlich verwurzelten Kundenstamm trifft, der in der näheren Umgebung wohnt und zunehmend seine täglichen Aktivitätsspektren, begründet durch verstärkte Heimarbeit als Folge der Pandemie und weniger Mobilität aufgrund der Energiekrise und Inflation, auf eben diese Bereiche ausrichtet. Damit werden Geschäftsstraßen zu gesellschaftlichen Treffpunkten, Flaniermeilen und Nachbarschaftszentren für die an sie grenzenden Kieze. Dadurch könnten solche innerstädtischen Geschäftsstraßen wie die Wilmersdorfer Straße (abermals) zu den Gewinnern gehören, während innerstädtische Geschäftsstraßen an reinen Arbeits- und Touristenstandorten und/oder nichtintegrierte Einzelhandelsstandorte (in der Pandemie noch als Gewinner aufgrund der Umgehungsmöglichkeiten von Kontakten durch motorisierten Individualverkehr) sich sicher etwas einfallen lassen müssen, um in einer (post‑)pandemischen Stadt weiterhin ihre Bedeutung zu erhalten.
